# Dorsal Root Ganglion Stimulation Therapy for Refractory Idiopathic Pudendal Neuralgia

**DOI:** 10.7759/cureus.34681

**Published:** 2023-02-06

**Authors:** Gaurav Chauhan, Suresh K Srinivasan, Suchit Khanduja

**Affiliations:** 1 Anesthesiology and Perioperative Medicine, University of Pittsburgh Medical Center Presbyterian, Pittsburgh, USA; 2 Pain Management, Trinity West Medical Center, Steubenville, USA; 3 Anesthesiology, Beaumont Hospital, Royal Oak, USA

**Keywords:** chat gpt, chronic pain, neuromodulation, dorsal root ganglion stimulation therapy, pudendal neuralgia

## Abstract

Dorsal root ganglion stimulation is a relatively new treatment option for chronic pain conditions such as pudendal neuralgia, which is a chronic pain condition affecting the pudendal nerve in the pelvic region. Pudendal neuralgia is a debilitating condition that can significantly affect the patient's quality of life. In dorsal root ganglion stimulation, a small device is implanted that delivers electrical impulses to the dorsal root ganglion to modulate pain signals coming from the pudendal nerve. The procedure is considered investigational and has been investigated in case series and case reports with promising results. However, more research is needed to fully understand its safety and effectiveness. This case report highlights the potential of dorsal root ganglion stimulation as a treatment option for pudendal neuralgia and the need for further research to establish it as a standard treatment option.

## Introduction

Pudendal neuralgia is a chronic pain condition affecting the pudendal nerve located in the pelvic region. The symptoms of pudendal neuralgia can include pain in the genital area, perineum, anus, and/or rectum, pain during sexual intercourse or ejaculation, pain when sitting for long periods, pain when standing up after sitting, pain when defecating or urinating, numbness or tingling in the genital area, and weakness or muscle spasms in the pelvic floor muscles. These symptoms can vary in intensity and may be intermittent or constant [1,2]. They can be triggered by certain activities, such as sitting, standing, or cycling, and can be accompanied by other symptoms, such as fatigue, depression, and anxiety. The incidence of pudendal neuralgia is not well established, as it is a relatively uncommon condition that is often misdiagnosed or overlooked. Studies have estimated the incidence to be between 0.5% and 2% of the population, with women being affected slightly more often than men. It is important to note that symptoms of pudendal neuralgia can be similar to other conditions, such as pelvic floor dysfunction, interstitial cystitis, prostatitis, or lumbosacral disc herniation [1-3].

The exact etiopathogenesis of pudendal neuralgia is not well understood. However, several factors have been identified that may contribute to its development. Trauma or injury to the pelvic region, such as injuries sustained during childbirth, surgery, cycling, or other physical activities that put pressure on the pelvic region, can cause pudendal neuralgia [3,4]. Compression of the pudendal nerve, as it passes through the greater sciatic foramen, the pudendal canal, or the Alcock’s canal, is caused by pelvic fractures, tumors, inflammatory conditions, or scar tissue, and can also lead to pudendal neuralgia [3,5]. Chronic pelvic muscle tension, overuse, or tension of the pelvic floor muscles can lead to chronic inflammation and irritation of the pudendal nerve. Neurological disorders, such as multiple sclerosis, spinal cord injuries, or diabetic neuropathy, can cause pudendal neuralgia. In some cases, the cause of pudendal neuralgia is unknown and is considered idiopathic [3-7].

The authors present a case of idiopathic pudendal neuralgia in a 35-year-old male patient refractory to conservative management, including medical management and pelvic rehabilitation. The patient underwent dorsal root ganglion stimulation therapy, which was successful in providing adequate symptom mitigation.

## Case presentation

A 35-year-old male patient that consented to this case report presented with severe pain in his inferior rectal, perineal, posterior scrotal, and penile region bilaterally. The patient reported that the pain started insidiously three months back and gradually worsened to the current intensity. The patient described the pain as burning, searing, knife-like, and stabbing pain and rated his pain as 8-10/10 on a numeric rating scale (NRS) for pain. The pain was intermittent, with multiple self-resolving daily episodes that lasted for a few minutes to a few hours. Patient-reported that putting himself in a lithotomy position would relieve the pain marginally. Besides a change in position to lithotomy, there were no other alleviating factors. He further noted that pain was exacerbated by sitting on hard surfaces, exercises such as squatting, and activities such as walking long distances and cycling. The patient reported no viral illness, significant or subtle trauma, or falls preceding the onset of his symptoms. The patient had 20 pack year history of smoking but no significant comorbidities. He also had no significant family history or psychological history. The patient worked as a painter for a construction company. He was on temporary disability due to his symptoms.

The patient was on Gabapentin 800 mg TID, Meloxicam 15 mg per day as needed for pain, and Oxycodone 10 mg four times daily as needed for severe pain during his first consult at the authors' pain clinic. The patient was also taking Fluoxetine 60 mg per day for anxiety and depression. The chronic pain psychologist diagnosed the patient with significant anxiety and depression. The state anxiety scale has a score range of 20-80, with a higher score depicting greater anxiety. The patient scored 68 on the state anxiety state scale. The patient scored 23 on Beck's depression inventory which translates into moderate to severe depression. The patient was on the current regimen for four months and reported sub-optimal pain control. Upon first visit to the authors' pain clinic, the patient was advised to switch Gabapentin to Pregabalin 150 mg three times per day and switch Meloxicam to Nabumetone 1000 mg twice daily as needed for pain. The patient was also advised to consult physical therapy and chronic pain psychology for pelvic rehabilitation and desensitization and chronic pain psychology cognitive behavior therapy. The patient underwent pelvic rehabilitation sessions twice weekly for three months focused on the pelvis, lower back, hips, and abdomen. It involved manual therapy, exercises, education, and modalities such as electrical stimulation or heat/cold therapy. The patient underwent pelvic floor muscle strengthening, core stability exercises, stretches, postural re-education, biofeedback, neuromuscular re-education, balance and coordination exercises. The patient underwent desensitization therapy during his pelvic rehabilitation sessions. Desensitization therapy was aimed to overcome their pain and fear response to specific movements and activities and eventually be able to perform these movements without discomfort. The patient also underwent Cognitive Behavior Therapy (CBT) to identify and modify negative thought patterns, reduce anxiety and stress, and improve coping skills. The patient underwent CBT in the form of weekly group therapy sessions.

**Figure 1:**
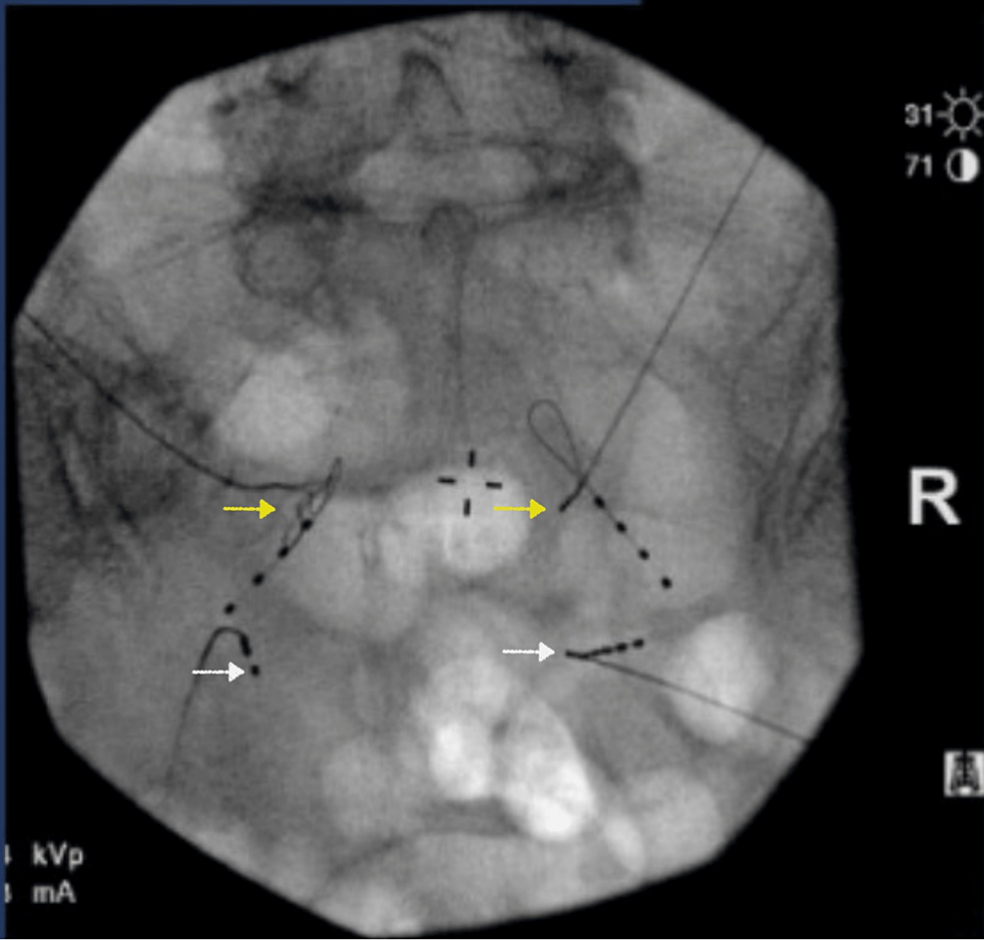
Anteroposterior fluoroscopic image of dorsal root ganglion stimulation leads placed bilaterally at S2 (yellow arrows) and S3 (white arrows).

**Figure 2:**
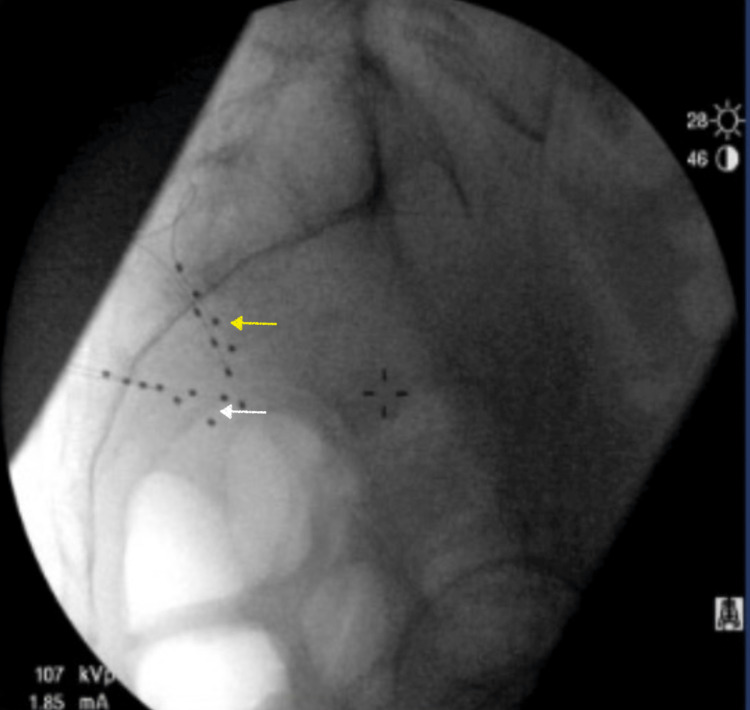
Lateral fluoroscopic image of dorsal root ganglion stimulation leads placed bilaterally at S2 (yellow arrow) and S3 (white arrow).

The patient was also scheduled for pelvic magnetic resonance imaging (MRI) and a diagnostic and therapeutic pudendal nerve block to discern the etiology further. The MRI didn't report any significant pelvic pathology or pudendal nerve compression. The patient underwent the bilateral diagnostic pudendal nerve block with 5 ccs of 0.25% Bupivacaine and 20 mg of Methylprednisolone acetate injected on each side. The pudendal nerve led to a complete resolution of his symptoms for four weeks, and the patient was assigned a definitive diagnosis of pudendal neuralgia. The patient then underwent pulsed radiofrequency ablation of both pudendal nerves under fluoroscopic guidance. The pulsed radiofrequency ablation failed to offer any relief beyond one week. At that point, the patient was also referred to pelvic rehabilitation and chronic pain psychology for desensitization and cognitive behavior therapy. The patient failed to continue pelvic rehabilitation and physical therapy due to his pain. At that point, the decision was made to offer the patient a trial of dorsal root ganglion stimulation (DRGS) therapy. The patient underwent a successful trial with the leads placed bilaterally at S2 and S3 foramen as can be seen in Figures 1, 2.

The patient reported 2-3/10 pain on the NRS for pain during the week of the trial. Subsequently, the patient underwent a successful implant of the DRGS leads, placed bilaterally at S2 and S3 foramen.

The patient is currently 11 months post-implant and is still reporting a significant improvement in his pain and associated symptoms. The patient was weaned-off opioids over a period 3-month post-implant and is currently on Pregabalin 50 mg BID. He is no longer using Nabumetone. The patient resumed his job three months after the DRGS system and is presently maintaining gainful employment.

## Discussion

The pudendal nerve is a mixed nerve that originates from the sacral plexus (S2-S4) and is responsible for the innervation of the pelvic region. It is comprised of both motor and sensory fibers. The motor fibers of the pudendal nerve supply the external urethral sphincter and the muscles of the perineum and anal sphincter. The sensory fibers of the pudendal nerve provide sensation to the perineum, the genitals, and the anus. The pudendal nerve travels through the greater sciatic foramen, where it divides into its three main branches: the dorsal nerve of the clitoris or penis, the perineal nerve, and the inferior rectal nerve. The dorsal nerve of the clitoris or penis supplies sensation to the glans of the clitoris or the penis. The perineal nerve supplies sensation to the perineum and the external anal sphincter. The inferior rectal nerve supplies sensation to the anus and the lower rectum. The pudendal nerve also has several communicating branches, which connect it to other nerves, such as the obturator nerve, the femoral nerve, and the sacral nerves, which allows it to have a broad distribution of innervation and sensation [6,7].

Presumptive diagnosis of pudendal neuralgia is guided by Nantes criterion. The criteria were developed by a group of French experts and are widely used to diagnose and classify pudendal neuralgia [8]. It is important to note that the Nantes criteria are not a definitive diagnostic tool, and it's not always easy to establish the diagnosis of pudendal neuralgia, as the symptoms can be similar to other conditions. In such a scenario, positive results on diagnostic tests, such as a pudendal nerve block or an electromyography test, can help to confirm the diagnosis [9,10]. Neuroimaging, including computerized tomographic or magnetic resonance imaging of pelvis and abdomen should be considered to evaluate for pudendal nerve compression, or etiologies that can mimic signs and symptoms of pudendal neuralgia. Imaging should definitely be done before surgical interventions [8,11].

Treatment options for pudendal neuralgia entail both non-surgical and surgical options. Non-surgical options include the use of medications such as non-steroidal anti-inflammatory drugs (NSAIDs), tricyclic antidepressants, and anticonvulsants which can help to reduce pain and inflammation [12]. There should also be enhanced focus on conservative methods such as pelvic rehabilitation, efforts to take pressure off the nerve, targeted physical therapy, and psychological techniques such as cognitive behavior therapy and biofeedback. Physical therapy can be helpful in improving circulation, reducing muscle tension, and promoting healing [11,13]. Injections of corticosteroids or local anesthetics can also be used to reduce inflammation and provide pain relief [14,15]. Additionally, lifestyle changes such as avoiding activities that can trigger symptoms, such as cycling and sitting for long periods, and maintaining a healthy weight can also be helpful in managing the condition. Other chronic pain interventions include pulsed radiofrequency ablation of the pudendal nerve. Two recent studies totaling 90 pudendal neuralgia patients treated with pulsed radiofrequency reported significant pain relief in 89% of those treated and followed for up to six months. Another study compared radiofrequency ablation with standard pudendal nerve blocks in 88 patients with pudendal neuralgia. It found rough equivalency in pain relief out to 30 days but improved symptoms with pulsed radiofrequency therapy extending from one to three months [16,17]. Analogous to radiofrequency ablation, cryotherapy of the pudendal nerve is also a promising modality, based on a recent case series, which reported an average reduction in pain of 60% for up to six months after the procedure [18]. However, there is lack of any larger prospective trials evaluating the long-term safety and efficacy of cryotherapy. Surgical options include pudendal nerve release, which aims to release the nerve from compression or entrapment and can be done by open or endoscopic surgery, and pudendal nerve decompression, which aims to relieve pressure on the nerve by removing surrounding tissue or bone that may be compressing it [19,20]. It is important to note that treatment options should be individualized and tailored to each person's specific needs, surgery should be considered as a last resort when non-surgical options have failed, and it is also important to work closely with a healthcare provider to monitor the effectiveness of treatment and adjust as needed [21].

Dorsal root ganglion stimulation (DRGS) therapy is a surgical procedure involving the implantation of a device that delivers electrical impulses to the spinal cord's dorsal root ganglion (DRG). This procedure has been investigated as a potential treatment option for pudendal neuralgia. This procedure involves the implantation of a small device that delivers electrical impulses to the sacral nerves (S2-S3) to modulate the afferent pain signals from the pudendal nerve. The device is implanted under the skin in the lower back and connected to leads placed near the sacral nerves. Depending on the patient's needs, the device can be programmed to deliver different electrical impulses, such as continuous or intermittent [22]. S2 and S3 neuromodulation is considered a less invasive alternative to traditional surgical procedures for pudendal neuralgia, such as nerve release or decompression. It is important to note that neuromodulation is not a cure for pudendal neuralgia, but it can help to reduce pain and improve quality of life. It is usually only recommended for patients who have not responded to conservative therapy or have failed surgical decompression for conditions such as chronic peripheral neuropathic pain, complex regional pain syndrome type I and II, failed back surgery syndrome and interstitial cystitis [23].

There have been several case reports of successful resolution of pudendal neuralgia with DRGS therapy. These case reports have shown that DRS therapy can provide significant pain relief for patients with pudendal neuralgia who have failed other treatments. A case report from 2016 reported successful use of neuromodulation leads, placed bilaterally at S2 and S3 foramina, for pudendal neuralgia refractory to conservative management [24]. Another case series from 2019 described chronic pelvic pain patient that failed conservative management but reported significant pain relief and improved quality of life after DRGS therapy [25].

DRGS is a complex surgical procedure and should be performed by a well-trained specialist. While it is considered a relatively safe procedure, it can have some potential complications like any surgical procedure. Some potential complications of DRGS therapy include infection at the surgical site, hematoma, which is a collection of blood outside of a blood vessel, device failure, lead migration, electrical interference with other electronic devices or medical equipment, implant-related pain, and adverse reaction to anesthesia [26]. The risk of complications can vary depending on the individual patient and the specific procedure. It is essential to work closely with a healthcare provider to monitor the effectiveness of treatment and address any complications that may occur. DRS therapy is a relatively new treatment option, and more research is needed to fully understand its safety and effectiveness [26,27].

## Conclusions

In conclusion, DRGS therapy is a promising treatment option for pudendal neuralgia. Dorsal root ganglion therapy can be added to the therapeutic algorithm of pudendal neuralgia refractory to conservative medical and surgical management. It is important for patients to weigh the potential benefits and risks of DRGS therapy with their chronic pain physician and to have realistic expectations about the outcomes of the procedure. Overall, DRGS therapy may be a viable treatment option for some individuals with pudendal neuralgia, but more research is needed to fully understand its safety and effectiveness.

## Figures and Tables

**Figure 3:**
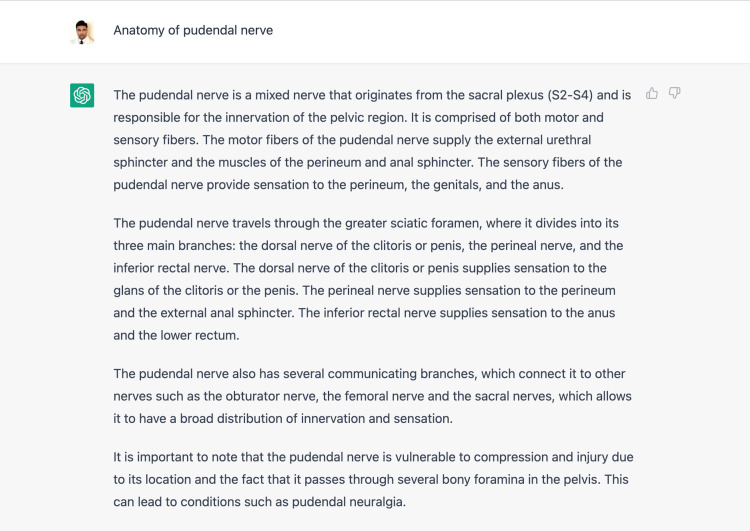
ChatGPT AI snapshot 1

**Figure 4:**
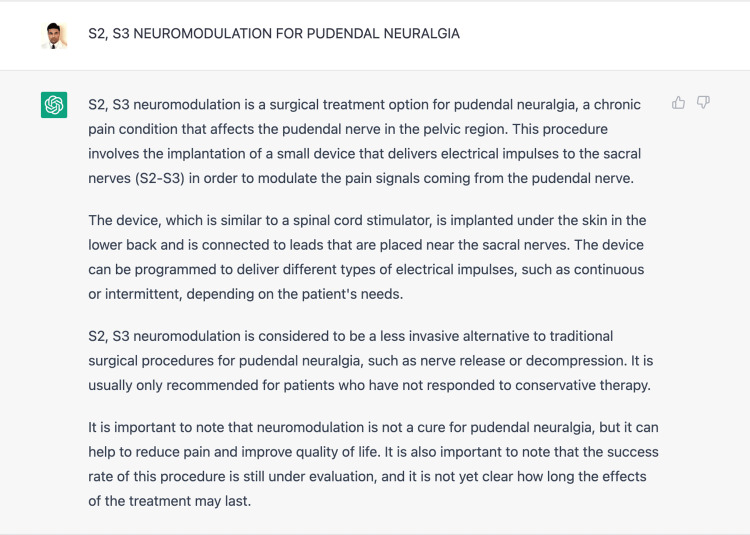
ChatGPT screenshot 2
